# Characterization of microglia behaviour in healthy and pathological conditions with image analysis tools

**DOI:** 10.1098/rsob.220200

**Published:** 2023-01-11

**Authors:** Aleix Martinez, Jean-Karim Hériché, Maria Calvo, Christian Tischer, Amaia Otxoa-de-Amezaga, Jordi Pedragosa, Anna Bosch, Anna M. Planas, Valérie Petegnief

**Affiliations:** ^1^ Institute for Bioengineering of Catalonia, 08028 Barcelona, Spain; ^2^ Cell Biology and Biophysics Unit, European Molecular Biology Laboratory, 69117 Heidelberg, Germany; ^3^ Advanced Optical Microscopy Facility, Scientific and Technological Centers. School of Medicine, University of Barcelona, 08036 Barcelona, Spain; ^4^ Centre for BioImage Analysis, European Molecular Biology Laboratory, 69117 Heidelberg, Germany; ^5^ Achucarro Basque Center for Neuroscience and Department of Neuroscience, University of the Basque Country UPV/EHU, Achucarro, 48940 Leioa, Spain; ^6^ Department of Neuroscience and Experimental Therapeutics, Institute for Biomedical Research of Barcelona, Spanish Research Council, 08036 Barcelona, Spain; ^7^ Institut d'Investigacions Biomèdiques Augustí Pi i Sunyer, 08036 Barcelona, Spain

**Keywords:** microglia, phagocytosis, morphometry, classification, image analysis, neuroinflammation

## Abstract

Microglia are very sensitive to changes in the environment and respond through morphological, functional and metabolic adaptations. To depict the modifications microglia undergo under healthy and pathological conditions, we developed free access image analysis scripts to quantify microglia morphologies and phagocytosis. Neuron–glia cultures, in which microglia express the reporter tdTomato, were exposed to excitotoxicity or excitotoxicity + inflammation and analysed 8 h later. Neuronal death was assessed by SYTOX staining of nucleus debris and phagocytosis was measured through the engulfment of SYTOX^+^ particles in microglia. We identified seven morphologies: round, hypertrophic, fried egg, bipolar and three ‘inflamed’ morphologies. We generated a classifier able to separate them and assign one of the seven classes to each microglia in sample images. In control cultures, round and hypertrophic morphologies were predominant. Excitotoxicity had a limited effect on the composition of the populations. By contrast, excitotoxicity + inflammation promoted an enrichment in inflamed morphologies and increased the percentage of phagocytosing microglia. Our data suggest that inflammation is critical to promote phenotypical changes in microglia. We also validated our tools for the segmentation of microglia in brain slices and performed morphometry with the obtained mask. Our method is versatile and useful to correlate microglia sub-populations and behaviour with environmental changes.

## Graphical abstract

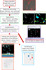

## 1. Introduction

Microglia are the immune cells of the brain. They constantly patrol the brain parenchyma to detect and destroy pathogens. Microglia are also involved in the maintenance of the homeostasis in the brain by the pruning of synapses, the control of neuronal activity and the contribution to oligodendrocyte survival [1].

In the damaged brain, microglia are activated by damage-associated molecular patterns (i.e. danger signals released by dying cells) [2]. Several studies support that microglia can play a detrimental role since they remove functional synapsis, secrete pro-inflammatory cytokines and nitric oxide that compromise neurons survival [3], and remove stressed but alive neurons in a process known as phagoptosis [4]. However, during the resolution of the inflammation, microglia play a critical role in the parenchyma repair since it engulfs and degrades cell debris and promotes the maturation of the precursors of oligodendrocytes [1]. Furthermore, activated microglia can protect neurons by synaptic stripping, a process that physically removes synaptic input [5].

Microglial cells are very plastic and they quickly respond to changes in the environment by adapting their function. Microglia are heterogeneous and several lines of evidence support that protective and detrimental microglia coexist in the same area in the injured brain, for example in animal models of stroke or spinal cord injury [6,7]. Therefore, the knowledge of whether the morphological diversity of microglia associates to specific functions may help to design therapeutic strategies to promote protection and repair. Recently, single-cell RNA-seq showed the existence of a sub-population termed disease-associated microglia (DAM) in a model of Alzheimer's disease that slows down the progression of the disease [8], but the putative correlation between the transcriptomic profile and the morphology has not been investigated. The genetic signature of DAM is similar to the genetic signature described in a sub-population of microglia found in early post-natal mice [9] and in a model of amyotrophic lateral sclerosis [8]. These DAM overexpress *Apoe, Tyrobp, Clec7a* and *Igf1* among other genes [9,10]. *Apoe* and *Clec7a* are involved in phagocytosis and *Clec7a* encodes the protein Dectin-1 which is expressed in ameboid proliferating microglia *in vivo* [9].

The heterogeneity of microglia has been reported in rat, mice and human brain under healthy and pathological conditions [11–17], and the number of publications describing methods to cluster microglia according to morphometric measurements *in vivo* has substantially increased in the last decade. Recently, Clarke *et al*. [15] calculated an inflammatory index based on morphometrics which was used to reflect a global state of inflammation or quantify changes in microglia ramifications. Although potentially useful to characterize morphologies present in cultures and in tissue sections, this pipeline does not provide a classification.

Though many studies are conducted *in vitro* to explore in depth microglia behaviour, very few address microglia morphological diversity in cell culture. The classification of microglia according to the morphology is challenging in cell culture because (i) differences in culture conditions (coating and culture media for example) and the presence of astrocytes and neurons affect microglia morphology, and (ii) classifiers are not available to discriminate all microglia sub-populations.

Since in the case of microglia, morphology is usually associated with function [18,19], we performed live cell imaging in primary cultures of neurons and glia and developed tools to characterize the behaviour of microglia exposed to different challenges. Live cell imaging is able to capture the transformation from a phenotype to another and enables combining morphometric measurements with functional assays. We compared morphology and phagocytic activity in control, excitotoxicity and excitotoxicity + inflammation-challenged cultures, conditions that mimic neuronal death and glial activation after cerebral ischaemia [20,21]. In addition, we validated the segmentation plugin to separate immunostained adult microglia in brain tissue sections after ischaemia. Altogether, we developed software to reliably segment and classify microglia according to morphology, with applications in cell culture systems and brain tissue sections. We consider our method as a starting point to conduct single-cell characterization.

## 2. Material and methods

### 2.1. Cell culture and treatments

Cultures were prepared using heterozygous embryos obtained from the crossing between homozygous B6.Cg-*Gt(ROSA)26Sor^tm9(CAG–tdTomato)Hze^*/J (#007909 JAX®Mice) mice with B6.129P2(C)-*Cx3cr1^tm2.1(cre/ERT2)Jung^*/J (#020940 JAX®Mice) mice, all in a C57BL/6J background. Pregnant females were deeply anaesthetized with 4% isoflurane and quickly euthanized by cerebral dislocation following a procedure approved by the ethical committees of the University of Barcelona and the Generalitat de Catalunya (OB282/18). We prepared mixed cultures containing microglia, astrocytes and neurons since they better preserve microglia identity compared to pure microglia cultures [22,23]. Eighteen-day-old embryos were extracted, decapitated and cortices were carefully removed and stripped of meninges. Cortical tissue was dissociated for 15 min in 0.05% trypsin/EDTA at 37°C followed by a mechanical dissociation in the presence of DNAse. Cells were plated at a density of 126 000 cells/well in a final volume of 300 µl in 5 µg ml^−1^ poly-L-lysine (SIGMA, cat. no. P-1524) pre-coated 8-well chambers Ibidi (Ibidi-Inycom, cat. no. 80827). Culture medium was MEM (SIGMA, cat. no. M4655) supplemented with 10% fetal bovine serum (Life Technology, cat. no. 16000-044) and 0.1 mg ml^−1^ gentamycin (Life Technology, cat. no. 15750-045). Culture medium was partly changed on 4, 7 and 10 days *in vitro* (DIV) and replaced with MEM + 2% B27 (Life Technology, cat. no. 17504-044) and gentamycin as described earlier [24]. On 10 DIV, 4-OH-Tamoxifen (SIGMA, cat. no. H7904) was added at 1 µM to trigger the expression of tdTomato protein in microglia. Lipopolysaccharide (LPS, SIGMA, cat. no. L8274) was added on 10 DIV at 1 µg ml^−1^ in some cultures to trigger inflammation.

On 12 DIV, medium was replaced with 300 µl of MEM + B27 and half of the wells were treated with N-Methyl-D-Aspartate (NMDA, SIGMA, cat. no. M3262) at 40 µM. One hour later, SYTOX™ Green Dead Cell Stain was added at 1 : 50 000 (ThermoFischer Scientific, cat. no. S34860) and chambers were placed in an incubation box at 37°C, 5% CO_2_ on the top of a Laser Scanning SP5 confocal microscope (Leica) equipped with a 20 x 0.7AN air objective. Images at 3 z planes were acquired with a zoom 2 every 4 min for 10 h. We usually acquired three fields/well and two–four wells/condition were analysed in each culture. The imaging conditions described in the study were defined as a compromise to characterize the microglia phenotypic change during excitotoxicity conditions and to preserve cell viability throughout the whole imaging experiment. Present conditions were tested in a previous study [25]. The temporal sampling rate (4 min) was set to resolve eventual transitions between the different microglia morphologies, lateral resolution (pixel size 758 nm) was set to define fine microglia processes and limits between touching cells and z sectioning interval (3z, 5–8 µm total thickness). This was useful to include in the analysis cells with different volumes and heights or cells that move over other cells such as prominent neurons clumps. This protocol was performed in all the cultures.

SYTOX staining in live cultures allows discriminating unlabelled live cells from labelled dying cells. SYTOX emits fluorescence after its binding to nucleic acids in cells with damaged membrane or to nucleic acids in cell debris.

### 2.2. Phagocytosis and cell death measurement

Engulfment/phagocytosis can be assayed using different methods: engulfment time *Δt* = *t*_end_ − *t*_start_ with *t*_start_ being the time of first contact and *t*_end_ being the time of membrane surrounding the target [26]; distance from the target and microglia branch retraction velocity [27]; percentage of microglia with engulfed debris [28]; and area fraction: area occupied by the target in the phagocyte [29]. We were interested in monitoring the phagocytosis of debris generated in the culture (i.e. with no addition of external cell fragments). Based on the last two methods, we developed the ImageJ macro PhagoSYTOX in order to detect all microglia and SYTOX^+^ particles using the appropriate thresholding method in ImageJ, usually Huang and IsoData, respectively. The macro generates maximum projection images obtained in the red channel, converts the image in a binary image and segments the microglia. An observer blind to the treatment checked the segmentation and manually edited when necessary. The segmentation of SYTOX^+^ particles is performed by the macro, and it usually requires no additional edition. Then, the macro generates a file containing the area of each microglia and of each particle and the list of particles outside and inside microglia cells. The observer checked for the presence of each particle inside the microglia in the corresponding time-lapse according to the following criteria: (i) the presence of phagosomes (pouches inside the microglia), (ii) microglia and the SYTOX^+^ particle move together over time and (iii) the size of the particle decreases over time. The macro is available from https://github.com/MariaCalvo-UB/Microglia_Phagocytosis.

Phagocytosis was calculated as follows in each frame of interest:

$$\frac{\left[100x(\mathrm{area}\;\mathrm{of}\;\mathrm{SYTOX}\;\mathrm{inside}\;\mathrm{microglia}/\mathrm{total}\;\mathrm{SYTOX}\;\mathrm{area})\right]}{\mathrm{total}\;\mathrm{number}\;\mathrm{of}\;\mathrm{microglia}}.$$

The number of phagocytosing microglia was determined by counting the cells in which the presence of at least one SYTOX^+^ particle was confirmed. Phagocytosing microglia was calculated as 100x(microglia with confirmed SYTOX^+^particle inside /total number of microglia). Basal SYTOX staining could not be measured because once the fields are selected in each well, we cannot open the chamber, add SYTOX and start the acquisition, so cell death progression was estimated between 4 h and 8 h post-NMDA and calculated as follows: Δ_SYTOX_ = area_t8h_-area_t4h_. We started to record the cells between 2.5 h and 4 h after the addition of NMDA. In a pilot experiment, we analysed changes in phagocytosis at 4 h, 8 h and 12 h and decided to perform the functional (phagocytosis) and morphological analyses at 8 h because preliminary results of phagocytosis (not shown) suggested a difference between control and NMDA-treated cells at this time point.

#### 2.2.1. Microtubule-associated protein 2 immunofluorescence

Cultures were fixed for 30 min with 4% paraformaldehyde at room temperature 24 h after NMDA addition. Cells were permeabilized for 5 min with 0.3% Triton X-100 in PBS, blocked for 30 min at RT in 3% normal goat serum in PBS and incubated overnight at 4% with an anti-microtubule-associated protein 2 (MAP2) antibody (1 : 3000; SIGMA, cat. no. M1406, RRID:AB_477171). After three washes, the cells were incubated for 1 h at RT with an Alexa Fluor 488 antibody (1 : 1000; Fisher Scientific, cat. no. A32723, RRID:AB_2633275). DAPI (Life Technologies, cat. no. D3571) was added at 300 nM and cells were incubated for 10 min to stain all nuclei. Images were acquired in an inverted fluorescent microscope Olympus IX71.

### 2.3. Image analysis, morphological feature extraction and classification

#### 2.3.1. Morphometric measurements: development of the ImageJ plugins

Maximum intensity projections of the red channel (561 nm) images to detect tdTomato were generated with ImageJ and saved. Plugins for segmentation, morphometry, results overlay and annotation were developed to complete this study and are available from the Fiji update site ‘Microglia Morphometry’. The source code, installation instructions and detailed descriptions of the analysis can be found online: https://github.com/embl-cba/microglia-morphometry#microglia-morphometry. A single frame or a sequence of frames can be analysed. In this study, we analysed the frame corresponding to the time 8 h post-NMDA. The plugin ‘New Microglia Segmentation and Tracking’ implements semi-automated segmentation of the cells. For this study, the segmentation parameters were set to *minimal cell size* = 200 µm^2^ and *maximal skeleton length* = 450 µm. A tunable relative threshold was incorporated to allow the segmentation of cells with faint signal, here we used *intensity threshold* = 1.5. Briefly, for automated segmentation, images are smoothed using an anisotropic diffusion filter and then binarized and converted to a label mask by connected component analysis. The generated mask can be manually edited to split touching cells or join unconnected parts of the same cell. A second plugin ‘Continue Microglia Segmentation and Tracking’ can be used to continue the edition of a label mask. The intensity and corresponding label mask images are then used to compute shape and intensity features for each segmented cell with the ‘Measure Microglia Morphometry’ plugin. Seventeen morphometric features are measured. In addition, the *ImageBoundaryContact_Pixel* feature counts the number of pixels touching the border of the image which allows the removal of incomplete cells in subsequent analyses to avoid misclassifying them. The plugin generates a table with the measured parameters that can be opened together with the mask with the plugin ‘Open dataset from table’ from the Fiji update site ‘Segmentation Annotator’ to identify individual cells and their corresponding measurements. With this plugin, a ‘Morphology’ column was added by the ‘Annotation’ command in order to manually annotate cells and associate them with a morphology label. The edited tables corresponding to images with annotated cells were saved and used for the training of the classifier.

#### 2.3.2. Analysis of the morphological cell populations

Further analysis was carried out using the R statistical software environment [30] using the UMAP implementation from the uwot package [31] and the HDBSCAN implementation from the dbscan package [32]. Ten additional morphological features were derived from some of the features generated above as follows:

Solidity = Area / ConvexArea
Roundness = Area / (EllipsoidLongestAxisRadius)^2^
Roundness2 = Area / (EllipsoidShortestAxisRadius)^2^
GeodesicElongation = (GeodesicDiameter)^2^ / Area
AspectRatio = (LargestInscribedCircleRadius)^2^ / Area
Circularity = Area / (Perimeter)^2^
Somaness = (RadiusAtBrightestPoint_Pixel)^2^ / Area
Branchiness = SkeletonNumBranchPoints / GeodesicDiameter
Straightness = (SkeletonLongestBranchLength)^2^ / Area
Thickness = Area / (SkeletonTotalLength)^2^

**Figure 1.**
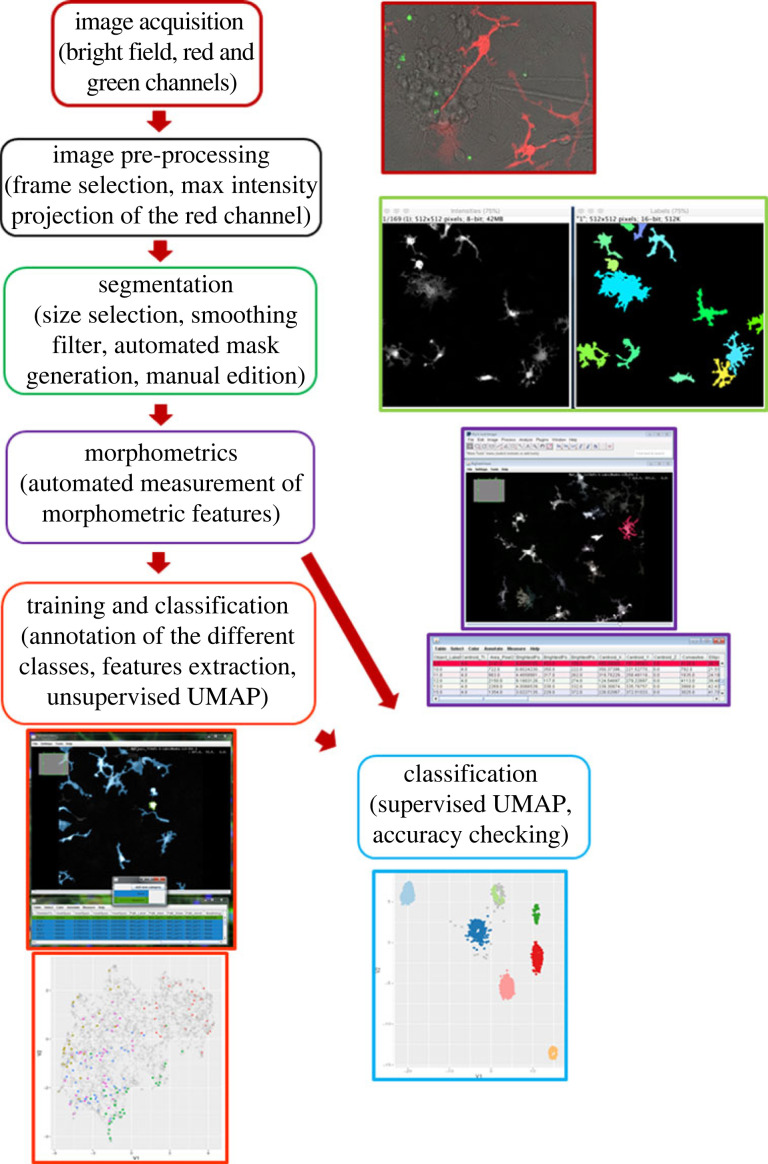
Workflow of the classification process of microglia in different morphologies. The main steps are indicated in separate frames and illustrated by the images and tables generated by the plugins.

Cells with a number of pixels in contact with the image border above 1% of their perimeter were considered incomplete and removed from the analysis. To classify all cells, we took a UMAP-based metric learning approach by taking advantage of some properties of the UMAP algorithm. In particular, UMAP can also be used in a supervised setting to make use of label information to find a mapping to a two-dimensional space in which points with different labels are separated while still preserving the underlying structure of the data. The mapping learned in the process can then be applied to new data points. With this approach points that are similar in the feature space end up in the vicinity of each other in the projection such that unannotated points will cluster with similar annotated ones. Conversely, points that are sufficiently dissimilar to the annotated ones are expected to fall into different regions. This process can be seen as a form of supervised feature engineering. We thus used annotated cells to learn a UMAP projection that separates the morphological classes and projected the unannotated cells in the resulting two-dimensional space. At this stage, no predictions are made about the unannotated points. To assign labels to all the points, applying a classifier is required. So, in a second step, we identified clusters in the projection using HDBSCAN and finally assigned a label to each cluster as a whole based on majority voting of the annotated cells in the cluster. The code used for this analysis is available in the accompanying computational notebook and in the repository at https://git.embl.de/heriche/microglia_morphology. A workflow with the main steps to achieve the classification of the cells is shown in figure 1.

The study included 16 cultures. For the phagocytosis measurement, two cultures were discarded for the following reasons. (i) In one time-lapse, images were not acquired at all time points for technical problems. Since we did not have the complete sequence, we were not able to quantify the phagocytosis but the morphometric study was possible. (ii) In another culture, NMDA did not cause cell death so no debris were found in the acquired fields and the phagocytosis could not be quantified. The phagocytosis analysis therefore includes 14 cultures. In the case of the morphology measurement, one culture was discarded because the intensity of fluorescence of tdTomato was too weak to allow an accurate morphometric analysis: 15 cultures were used. Therefore, in 13 cultures, we evaluated both phagocytosis and morphometry.

### 2.4. Statistical analysis

To compare groups, one-way ANOVA followed by the Tukey's multiple comparison test was used for death and the Kruskal–Wallis test was used for engulfment after testing for normality with GraphPad Prism8. ^#^*p* < 0.0001, ***p < 0.001, **p < 0.01 and *p < 0.05 versus control. Chi-squared tests were used to analyse the proportion of phagocytosing microglia and to compare the effect of treatments. **adj.*p*.value < 0.01, ^#^adj.*p*.value < 0.0001.

## 3. Results

### 3.1. Excitotoxicity recruits more microglia to engulf nuclear debris

**Figure 2.**
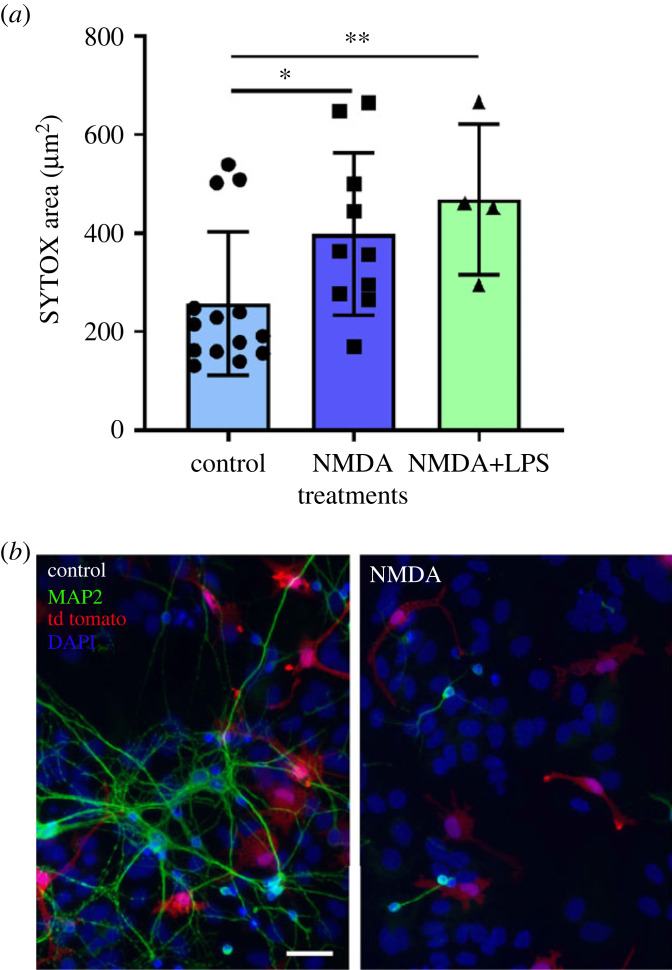
Cell death measurement. (*a*) Quantification of nuclei staining with SYTOX-green between 4 and 8 h post-NMDA. Results are expressed as mean ± s.d. (*n* = 4–14 independent cultures). *p < 0.05 and **p < 0.01 indicate a significant difference when compared to control. (*b*) MAP2 immunofluorescence (in green) and DAPI staining at 24 h post-NMDA. Scale bar = 50 µm.

Excitotoxicity is the main cause of neuronal death after an acute ischaemic lesion and overstimulation of glutamatergic NMDA receptors is widely used to mimic ischaemia *in vitro* [20]. We have previously characterized NMDA-induced cell death at 24 h in mixed neuron–glia cultures [33]. Here we performed time-lapse microscopy from 4 h to 14 h post-NMDA since we wanted to capture early changes in microglia behaviour in this model. We used SYTOX to quantify cell debris in our cultures as an indicator of cell death. Some cell death occurs in control cultures, and as expected, NMDA induced a significant increase in cell debris (SYTOX^+^ particles; figure 2*a*) at 8 h. The loss of MAP2 immunofluorescence at 24 h post-NMDA confirmed NMDA toxicity (figure 2*b*).

**Figure 3.**
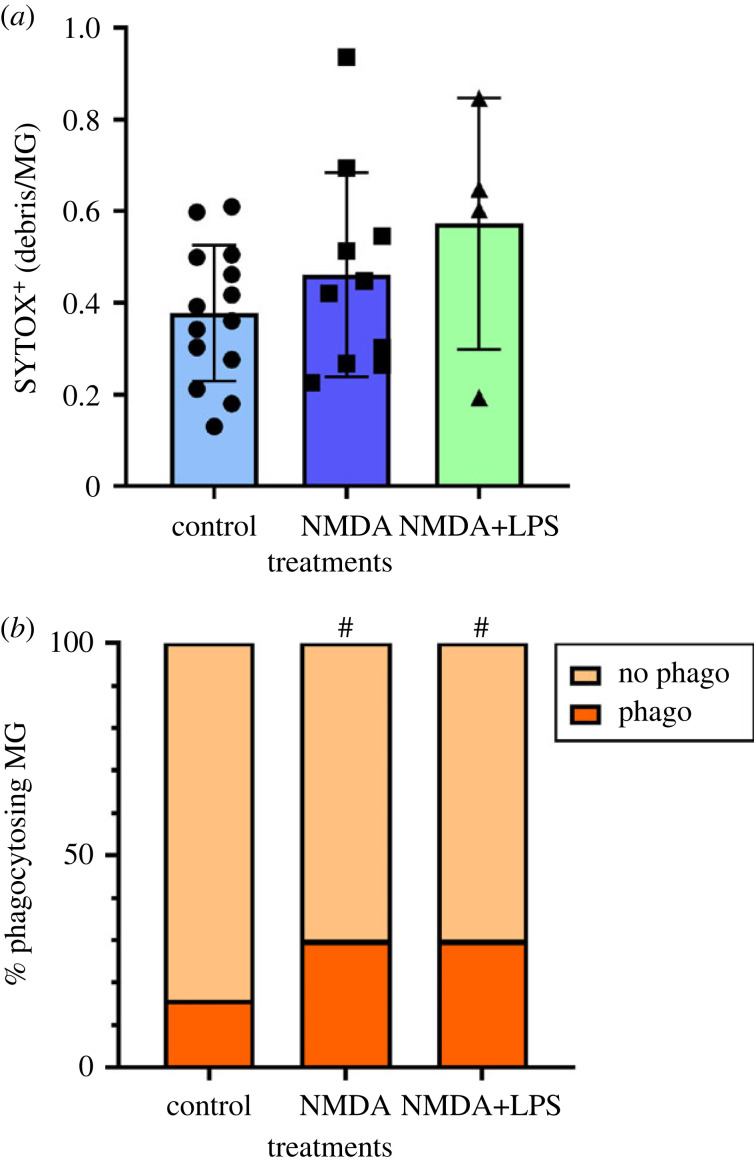
Phagocytosis after NMDA and NMDA + LPS. (*a*) The area of SYTOX^+^ particles inside microglia are quantified and normalized to the number of microglia per field. Results are expressed as mean ± s.d. (*n* = 4–14 independent cultures). (*b*) The proportion of phagocytosing microglia (containing at least one SYTOX^+^ particle) is calculated in each field. ^#^*p* < 0.0001 indicates a significant difference when compared to control.

In addition, we used SYTOX staining to monitor the phagocytosis of cell debris by microglia. In this assay, we monitored the engulfment of cells debris produced by natural and excitotoxity-induced cell death in primary cultures. We observed that phagocytosis of nuclear cell debris is infrequent in control mixed neuron–glia cultures. Eight hours after the addition of NMDA or NMDA + LPS, we observed no change in the amount of particles engulfed when compared to control (figure 3*a*). NMDA and NMDA + LPS induced cell death and production of more debris (total area of SYTOX + particles), and we calculated the index of phagocytosis taking into account the total area occupied by cell debris. Since the amount of engulfed debris was not increased after both pathological treatments, this means that microglia phagocytosed the same proportion of debris from a total amount in control and pathological conditions. Therefore, our data suggest that microglia in control and pathological conditions are equally efficient in phagocytosing nuclear debris.

The fact that the proportion of phagocytosing microglia was significantly higher in both pathological treatments when compared to control (30% versus 16%, figure 3*b*) suggests that increasing the number of phagocytosing microglia is a mechanism to deal with an increased accumulation of cell debris.

### 3.2. Microglial cells morphologies and classification

**Figure 4.**
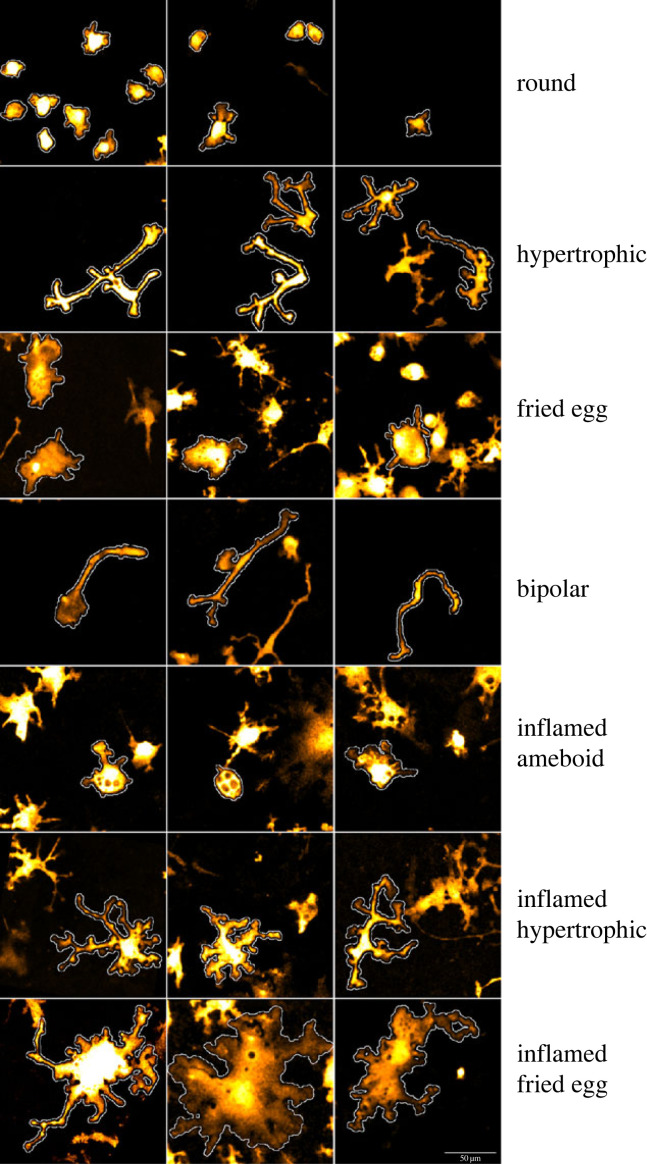
Representative confocal images of the seven microglia morphologies observed in the cultures. Scale bar = 50 µm.

We initially defined four morphological classes of cells that we named: round, fried egg, hypertrophic and bipolar (figure 4, representative examples). Round microglia are small cells lacking processes and have been considered immature microglia [34]. Fried egg microglia display a large cytoplasm, no processes, are quite static and contain few small cell debris. Bipolar, also called rod-like microglia, have a central nucleus and two long and thin unbranched processes. They have been described in healthy and pathological human brain [16] but their role is still unknown. Hypertrophic are quite heterogeneous, usually with a rectangular cytoplasm, several processes and few branching.

In addition, particularly after LPS treatment, we identified cells with a noticeably distinct morphology that we termed ‘inflamed’ phenotype. Inflamed cells generally appear thicker with a larger cytoplasm with more/longer branches (figure 4). Therefore, we further subdivided the classes resulting in a total of seven morphological classes: round, inflamed ameboid, fried egg, inflamed fried egg, hypertrophic, inflamed hypertrophic and bipolar. We annotated 379 cells into one of these seven classes across seven cultures and used them for training. A total of 4861 cells, including the annotated ones, were classified.

**Figure 5.**
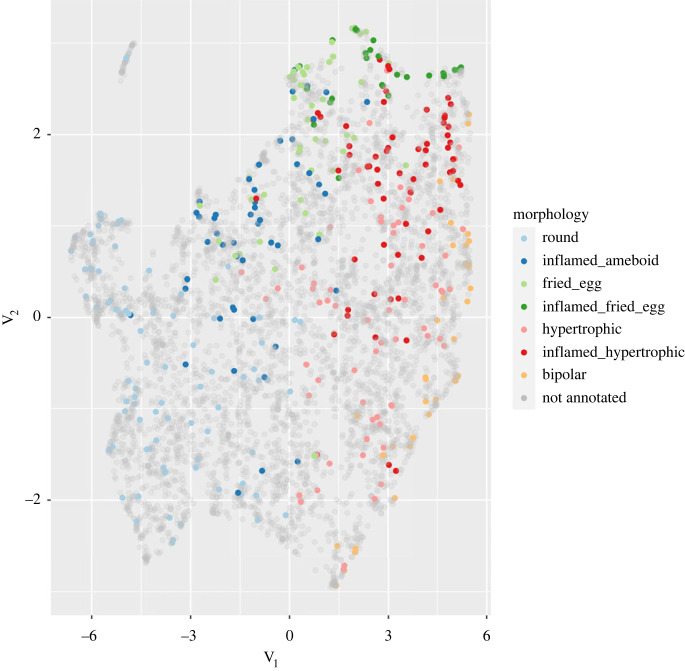
Unsupervised projection in a two-dimensional space of annotated and unannotated microglia.

**Figure 6.**
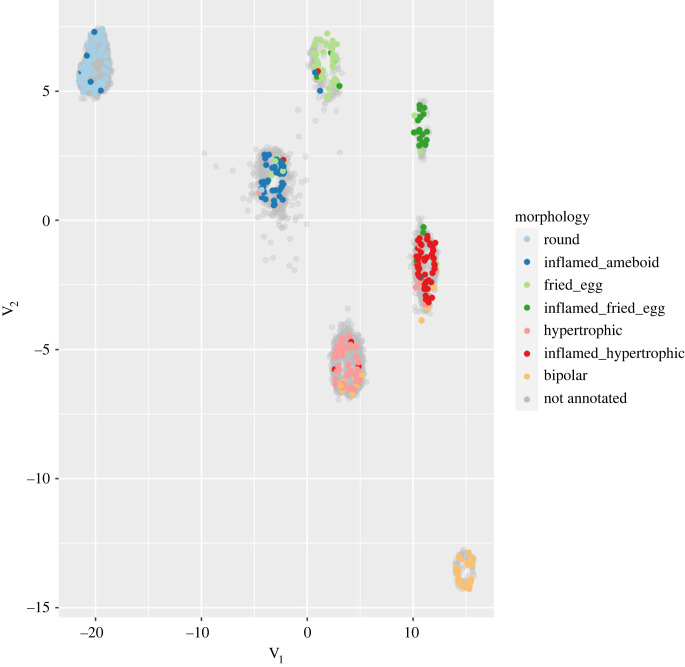
Supervised projection. Annotated cells were used to learn a two-dimensional space where the classes are separated; then unannotated cells were projected in this space. Most unannotated cells fall within clusters defined by the annotated cells.

**Figure 7.**
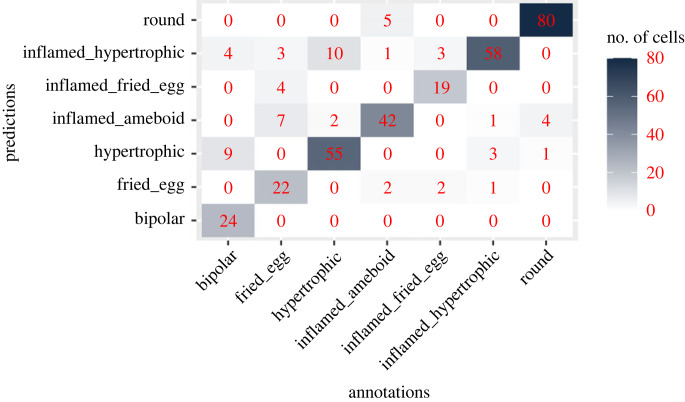
Confusion matrix showing classifier performance for the different classes.

To visualize whether the morphological feature vectors computed above can capture differences between the classes, we applied the UMAP dimensionality reduction method [35] to project the cells into a two-dimensional space. Plotting cells along these two dimensions shows that annotated cells of different classes generally occupy different regions (figure 5) indicating that the features distinguish differences between the morphological classes we identified. To obtain a better separation, we took a supervised approach in which we used the annotated cells to learn a UMAP projection that separates the classes (figure 6). Unannotated cells were then projected into this new space and fell essentially within the regions defined by the classes, indicating that our classes cover almost all the morphological diversity present in the cultures. To classify unannotated cells, we first identified groups of cells in the projection using the HDBSCAN algorithm [36] and assigned to each cell the label of the class most represented in its cluster. Fivefold cross-validation of this classifier indicates an overall classification accuracy of 82% (figure 7) with only 2% of the cells remaining unassigned. An observer blind to the assignments manually checked for the morphology of randomly selected microglia and disagreed with the predictions in 9.5% of the cases. The mismatch between the human-assigned label and the computer-generated one can be attributed to microglia with a morphology intermediate between two classes due to its displacement, a transient interaction with another cell or the extension of a process for a cell debris uptake.

### 3.3. Effect of treatment on morphological microglia populations

In our control neuron–glia cultures, the most abundant microglia populations are round and hypertrophic (electronic supplementary material, movie S1), that account for 37% and 30% of the total morphologies, respectively. We observed that they are the main phenotypes able to engulf cell debris, round being the most phagocytic phenotype. Regarding microglia movements, round microglia seem to roll on themselves and their displacement is very small (electronic supplementary material, movie S1). Bipolar are the fast-moving microglia and they move over comparatively longer distances (electronic supplementary material, movie S2). Fried egg microglia have not been described *in vivo* but they were reported in mixed glia cultures treated with LPS or LPS + INF*γ* [37]. We did not detect the typical cup structures of phagocytic cells in this phenotype (electronic supplementary material, movie S3), whereas cups are clearly present in round and hypertrophic microglia (electronic supplementary material, movie S4).

**Figure 8.**
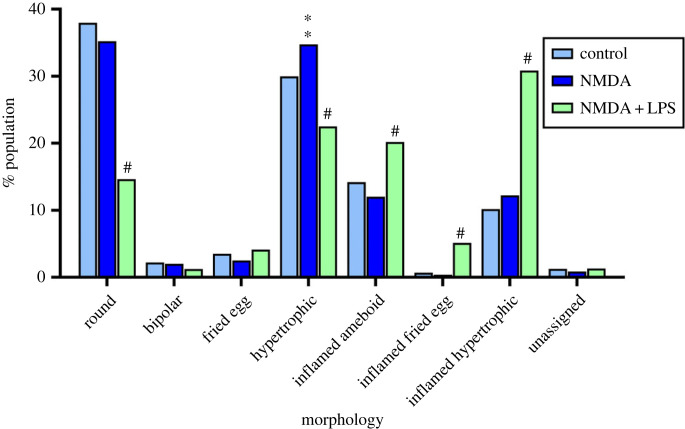
Microglia composition in the different morphological populations in control, NMDA and NMDA + LPS-treated cultures. Excitotoxicity + inflamation induced a dramatic change in the distribution of the microglia populations (*n* = 5–15 cultures). **adj.*p*. < 0.01, ^#^adj.*p*. < 0.0001 indicate a significant difference when compared to control.

We next examined how the composition of the microglia population was affected by different environments. The addition of NMDA alone slightly but significantly increased the proportion of hypertrophic microglia when compared to vehicle-treated cultures (electronic supplementary material, movie S5, figure 8). However, the combined NMDA + LPS treatment elicited a strong increase in the fraction of cells in the inflamed hypertrophic class and to a lesser extent in the inflamed ameboid class whereas it strongly decreased the proportion of round and hypertrophic microglia (electronic supplementary material, movie S6; figure 8). In addition, the proportion of inflamed fried egg rose from below 1% in control cultures to 5% after the NMDA + LPS treatment.

In addition to the cell culture application, we explored whether our segmentation plugin was able to identify microglia in brain tissue sections immuno-labelled with an anti-P2RY12 antibody. In our mouse model of ischaemia/reperfusion, microglia are highly branched with large area and perimeter in the healthy contralateral hemisphere (electronic supplementary material, figure S1). By contrast, at the periphery of the lesion and in the ischaemic core, microglia are smaller, with a higher index of circularity and solidity, as we previously showed in a model of permanent ischaemia [25] (electronic supplementary material, figure S1). Using the same workflow as for microglia in culture, we first performed an unsupervised UMAP (electronic supplementary material, figure S2A) and as expected, analysis showed that microglial cells in the contralateral non-ischaemic hemisphere, in the periphery of infarction and in the core of infarction display different morphologies. We then identified and annotated four morphologies (electronic supplementary material, figure S2B) in the brain tissue: (a) highly ramified with a round expansion area; (b) elongated ramified with a rectangular/elliptic expansion area; (c) with some similarity to the inflamed hypertrophic observed in culture; and (d) similar to inflamed ameboid present in culture. We performed morphometric measurements with the Microglia Morphometry plugin. With the supervised analysis of the clusters (electronic supplementary material, figure S2C), we were able to separate the four morphologies and their location in the three brain regions (electronic supplementary material, figure S2D). More importantly, the distribution pattern of the different microglia populations was statistically different when we compared the healthy hemisphere with the periphery and the core of the infarct (electronic supplementary material, figure S2D). The ramified forms (a + b) represent 68.9% of the microglia in the contralateral hemisphere versus 2.4% in the periphery and 1.4% in the core. In the periphery of the infarct, the predominant morphology displays an enlarged soma with short processes (c), whereas in the core, the inflamed ameboid morphology (d) is the most abundant.

## 4. Discussion

Due to the multifaceted nature of microglia, it is fundamental to properly depict and understand microglia heterogeneity and its biological meaning. Live cell image analysis is very useful to demonstrate the association between morphology and function and it reflects the interaction of microglia with other cell types and the surrounding milieu. Though imaging methods become more and more sophisticated to provide accurate data, we need reliable and user-friendly tools to analyse cell phenotype. For this reason, we present here a package of tools usable with primary neuron–glia cultures under control and pathological conditions, and with some applicability in histological tissue sections. In time-lapse fluorescence microscopy movies, we were able to analyse at the same time phagocytosis and decompose the cell population into its microglia subtypes. Our quantitative approach is able to detect differences between healthy and pathological conditions.

Usually, phagocytic assays employ (i) 1–3 µm fluorescent microspheres of bare latex or of polystyrene (ii) beads conjugated with zymosan, a component of the *Saccharomyces cerevisiae* wall [25], bacteria (*Staphylococcus aureus* or *Escherichia coli*) or (iii) isolated stained cells [25,28,38]. By contrast, our culture conditions that allow the growth of neurons, astrocytes, microglia from day 0 *in vitro*, obviate the need to seed neurons on the top of microglia or vice-versa to monitor phagocytosis of dead neurons. Neuronal cell death naturally occurs at low level in our mature primary cultures. Nucleic acid debris generated in the culture were stained with SYTOX, a fluorescent intercalating agent, and we quantified the engulfment of SYTOX^+^ particles by microglia. Since microglia express tdTomato, we were able to monitor specifically the uptake of green SYTOX^+^ particles in red microglia. This method may better mimic a physiological process than the engulfment by microglia of coated beads [39] or of apoptotic cells obtained from a separate culture [28]. To our knowledge, it is the first time that such a method has been reported in cell cultures. NMDA and NMDA + LPS did not cause a widespread phagocytosis of cell debris by microglia (as measured by SYTOX^+^ particles inside microglia over total SYTOX^+^ particles) at 8 h when compared to control. However, excitotoxicity and excitotoxicity + inflammation significantly increased the proportion of phagocytosing microglia from 16% in control to 30% after treatment, suggesting the recruitment of more microglial cells for the phagocytic activity to cope with the increased need of removing cell debris (figure 3).

The ubiquitous expression of the tdTomato reporter in microglia allowed a morphometric study. The use of embryo-derived cells with a culture medium optimized for the survival of neurons (low FBS concentration) results in a low density of microglia that grow in clones with limited interactions between clones in our cultures. Though *in vitro* cultures are supposed to provide a controlled environment, primary cultures of neuron–glia do not allow the formation of a uniform microglia layer and this may affect microglia morphology. Our primary cultures contain four out of the five morphologies that have been described in the hippocampus of human post-mortem brain [40]. In addition, Szabo & Gulya [39] described morphologies similar to our round and hypertrophic microglia in mixed rat cultures at 14 DIV (figure 4). Although the proportion of hypertrophic microglia increased significantly from 30 to 35% after NMDA, we were not able to associate excitotoxicity with a striking change in the composition of the microglia morphologies when compared to control (figure 8). This suggests that microglia did not receive enough signals to stimulate the P2RY12 receptor and to support the transformation from ramified/hypertrophic to ameboid phagocytic microglia described in the literature [41]. Hypertrophic microglia surround β-amyloid plaques in animal models of Alzheimer's disease and Bennett & Viaene [42] suggested that they could belong to the DAM cluster. The lack of a marked morphology change in the first hours after NMDA treatment suggests some blockade of the activation. Different reasons could account for this effect (e.g. the high density of healthy neurons and astrocytes may prevent a strong microglia reaction in the early time points after NMDA treatment). Moreover, maturation stage and intensity of the lesion may also explain differences in the extent of phenotypical changes in microglia. For example, Perez-Capotte *et al.* [37] reported the transformation of small round into large ameboid-shaped microglia 24 h after an injury with 100 µM glutamate in neuron–glia cultures of cerebellum prepared from 7-day rat pups, whereas we used mouse cortex from 18-day-old embryos.

By contrast, the addition of LPS together with NMDA induced a prominent change in the composition of the microglia cell population (figure 8). We did not clearly observe a gradual transformation from one morphology into another in our time-lapse study, although round and inflamed ameboid, fried egg and inflamed fried egg, hypertrophic and inflamed hypertrophic share common morphological traits (figure 4). While the external shape was similar between an inflamed morphology and its cognate non-inflamed type, the cytoplasm was much larger in the former. In the case of the comparison between inflamed hypertrophic and hypertrophic, the processes of the former were usually longer and more ramified. Interestingly, Szabo & Gulya [39] described in mixed rat cultures at 21–28 DIV (old cultures) the presence of microglia with long branches and a large soma that look similar to the inflamed hypertrophic microglia we observed mainly after NMDA + LPS treatment. Having established a method that allows the characterization and quantification of the microglia sub-populations in unstimulated and pathologic situations, it would be interesting to unravel the role of the predominant inflamed hypertrophic morphology and clarify whether its presence reflects the *in vivo* high state of inflammation, a senescence-like process in microglia or it corresponds to the expansion of microglia due to the reduction of the neuronal population caused by ageing.

The downregulation of homeostatic genes such as P2RY12 has been described after inflammatory conditions that occur for instance after stroke [43]. In the present study, using the tunable threshold of the New Microglia Segmentation and Tracking plugin, we were able to identify faintly P2RY12-immunostained microglia 4 days after ischaemia and perform morphometric studies, suggesting that this plugin is useful for the analysis of adult microglia after immunostaining. The Microglia Morphometry plugin was run on the labelMasks of the tissue sections and as in the cell cultures, the study enabled the identification and classification of various morphologies and showed a different distribution pattern in the non-ischaemic hemisphere, the peri-infarct region and the core of the infarct. Though, a potential limitation of the study is that a two-dimensional projection could underestimate the microglia branching. A recent study [14] classified microglia from brain slices immunostained with Iba-1 at 24 h reperfusion in a model of transient ischaemia in mice and identified four classes of microglia (ramified, rod-like, activated and amoeboid), and their distribution was quantified in the cortex and hippocampus in the control and infarcted regions. However, in this study, the authors did not discriminate between periphery and core. Despite the differences in the time post-lesion (24 h versus 4 days in our study), brain regions (cortex-hippocampus versus cortex-striatum) and the severity of the lesion, the conclusions are similar to ours: there is a significant loss of the ramified morphologies and an increase in the activated phenotypes (activated and amoeboid) in the affected hemisphere when compared to the healthy hemisphere.

In the course of this study, we developed new tools that are useful on their own or in combination with elements of the now growing ecosystem of microglia analysis tools. In particular, we want to highlight our ‘Segmentation Annotator’ plugin which is an essential, easy-to-use tool for building a classifier training set.

With the computational tools we developed, we showed that:

(i) Excitotoxic neuronal death recruits a higher number of microglia for the phagocytosis.
(ii) The composition of the different microglia morphologies is associated with the activation status.
(iii) Neuronal cell death in the absence of inflammation does not promote a morphological transformation.
(iv) Neuronal cell death and inflammation produce an enrichment of inflamed morphologies.

## 5. Conclusion and future perspectives

We generated semi-automated user-friendly programs for ImageJ that allow quantifying cell debris engulfment and sub-populations of microglia, and that can be very helpful to discriminate microglia clusters when exposed to different environments. The software is publicly accessible to investigate microglia morphology in different model systems (cultures and tissue sections). To our knowledge, our study is the first to provide tools that classify microglia morphologies *in vitro*. These tools are potentially useful to study macrophages, genetically modified microglia and microglia/macrophages response to anti-inflammatory treatments.

## Data Availability

Data that support the morphological findings of this study are available from the public repository BioImage Archive (S-BIAD401) and the scripts for the morphometric studies and phagocytosis are available from https://github.com/embl-cba/microglia-morphometry and https://github.com/MariaCalvo-UB/Microglia_Phagocytosis. The code used for the microglia morphometry is available from https://git.embl.de/heriche/microglia_morphology. In addition, our morphometric feature data can be interactively explored and visualized with the Image Data Explorer as described here: https://git.embl.de/heriche/microglia_morphology#to-explore-the-data-with-the-image-data-explorer. Additional data are available from the corresponding authors upon reasonable request. Movies and additional information are provided in the electronic supplementary material [44].
